# Understanding Pediatric Bacterial Preseptal and Orbital Cellulitis

**DOI:** 10.4103/0974-9233.63074

**Published:** 2010

**Authors:** Mithra O. Gonzalez, Vikram D. Durairaj

**Affiliations:** Department of Ophthalmology, Division of Oculoplastic and Orbital Surgery, Rocky Mountain Lions Eye Institute, University of Colorado, Aurora, CO, USA

**Keywords:** Eyelid Abscess, Orbit Infection, Orbital Abscess, Orbital Cellulitis, Preseptal Cellulitis

## Abstract

Pediatric preseptal and orbital cellulitis are infectious disorders that result in periorbital inflammation. Preseptal cellulitis is often associated with breaches in the skin barrier whereas orbital cellulitis is commonly associated with paranasal sinusitis. Orbital cellulitis may be associated with subperiosteal abscess. It is important to distinguish between preseptal from orbital cellulitis. Clinical examination and diagnostic imaging are useful in determining appropriate management. Patients are usually treated with broad spectrum antibiotics and surgery when indicated.

## INTRODUCTION

Pediatric preseptal or orbital cellulitis may develop from either contiguous extension from periorbital structures or from both exogenous or endogenous sources.^1^ Breaches in the skin barrier such as trauma and insect bites may be more commonly associated with preseptal cellulitis than orbital cellulitis.^2^ Orbital cellulitis is highly associated with paranasal sinusitis.^1^–^3^ Ethmoidal sinusitis has been reported in 84–100% of cases of orbital cellulitis.^1^–^3^ This is especially true of the relatively rare cases of infantile orbital cellulitis and its exceedingly rare counterpart, neonatal orbital cellulitis.^4^,^5^

**Figure 1:**
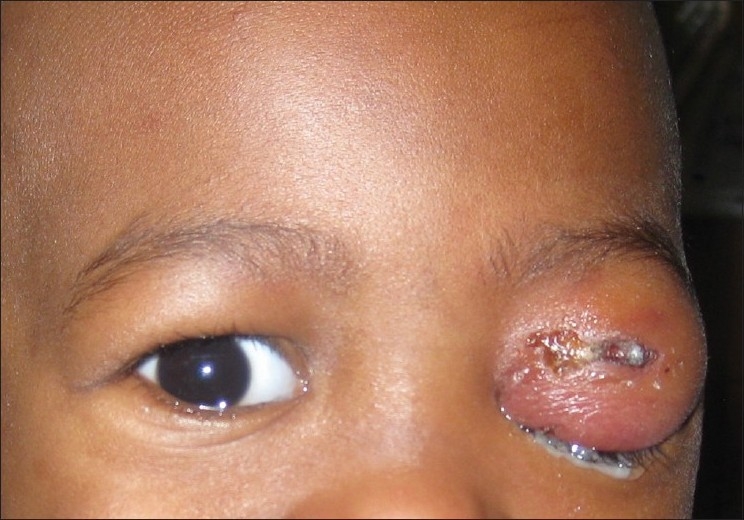
Left orbital cellulitis secondary to penetrating dog bite

The role of ethmoidal sinusitis in orbital cellulitis has been the cause of much speculation. The medial wall of the orbit is the thinnest and most porous of the orbit and may account for the contiguous extension.^1^,^6^ Furthermore, a shared valveless venous system has been cited as a possible means of spread.^1^,^6^ Dental procedures, blunt trauma, penetrating trauma, orbital, and periorbital surgery constitute some of the exogenous causes of orbital cellulitis^1^,^7^ [Figure 1]. Endogenous causes may include sepsis and endophthalmitis.^1^

## CLASSIFICATION OF PERIORBITAL AND ORBITAL CELLULITIS

Historically, Chandler's classification of orbital complications of acute sinusitis has been used.^8^

### Chandler classification

Group 1: Preseptal cellulitis

Group 2: Orbital cellulitis

Group 3: Subperiosteal abscess

Group 4: Intraorbital abscess

Group 5: Cavernous sinus thrombosis

Jain and Rubin recently simplified the classification system^1^

### Jain/Rubin classification

1. Preseptal cellulitis
2. Orbital cellulitis (with or without intracranial complications)
3. Orbital abscess (with or without intracranial complications)
  - Intraorbital abscess, which may arise from collection of purulent material in an orbital cellulitis
  - Subperiosteal abscess, which may lead to true infection of orbital soft tissues

Bacterial orbital cellulitis consists of inflammation caused by a bacterial infection located in the postseptal space. The postseptal process may result in orbital signs that may include exophthalmos/proptosis (61–99%) and diplopia (46–54%).^2^,^3^ Pain, restricted motility and vision loss may also be the presenting signs of orbital cellulitis. One should also note that the signs and symptoms of acute sinusitis may also commonly present simultaneously.^2^

Vision loss may result from insult to the optic nerve or retina through a variety of mechanisms including orbital compartment syndrome, vascular infiltration, mass effect, and optic neuritis.^9^,^10^

## PATHOGENS OF PEDIATRIC BACTERIAL PRESEPTAL AND ORBITAL CELLULITIS

Prior to 1985, *Haemophilus influenza* (*H. flu*), was not only a feared pathogen of preseptal cellulitis, it was also the most common causative pathogen of orbital cellulitis.^11^ Bacteremia was found in up to 80% of children with periorbital cellulitis.^12^,^13^ *H. flu* is a virulent pathogen that has been associated with meningitis, cavernous sinus thrombosis and death.^14^,^15^ As a result, prior to 1985, many infectious disease experts recommended cerebrospinal fluid (CSF) analysis and culture in children with periorbital cellulitis.^12^,^15^ In 1985, the *H. flu* Type B vaccine was introduced and changed the microbiological spectrum of bacterial periorbital cellulitis.^11^,^13^,^15^ As more children became vaccinated, the incidence of pediatric *H. flu* periorbital cellulitis decreased and the total number of *H. flu* infections decreased.^11^,^13^,^15^ Recent retrospective studies have confirmed this decreasing trend in *H. flu* periorbital cellulitis.^2^,^3^ Currently, *Staphylococcus aureus* and *Streptococcus* species cause the majority of culture positive cases of preseptal or orbital cellulitis.^2^,^3^,^15^

*Staphylococcus aureus* has been found to be remarkable pathogen because of the increasing frequency of Methicillin-resistant *Staphylococcus* *aureus* (MRSA) in all types of infections including periorbital infections.^16^ Infantile and neonatal orbital cellulitis is commonly associated with *S. aureus* positive cultures.^4^,^5^

## DIFFERENTIAL DIAGNOSIS

After a preseptal process has been ruled-out by the presence of orbital signs, the differential diagnosis of bacterial orbital cellulitis includes other causes of orbital inflammation. Specifically, mycotic orbital celluitis, neoplasm, thyroid eye disease, and idiopathic orbital inflammation as well as autoimmune, congenital, and traumatic disease should be considered in the diagnosis of bacterial orbital cellulitis.

## DIAGNOSTIC STUDIES

Once a bacterial orbital cellulitis is diagnosed or suspected, the child may benefit from additional diagnostic studies. Blood work may include a complete blood count (CBC) and blood cultures. Although the diagnostic yield of blood cultures is low compared to that of surgical aspirates, a positive result may help tailor antibiotic therapy.^3^,^11^ CSF analysis is no longer routinely ordered with the exception of bilateral cases of orbital cellulitis where meningitis and/or intracranial involvement is suspected.^1^,^13^,^16^

**Figure 2:**
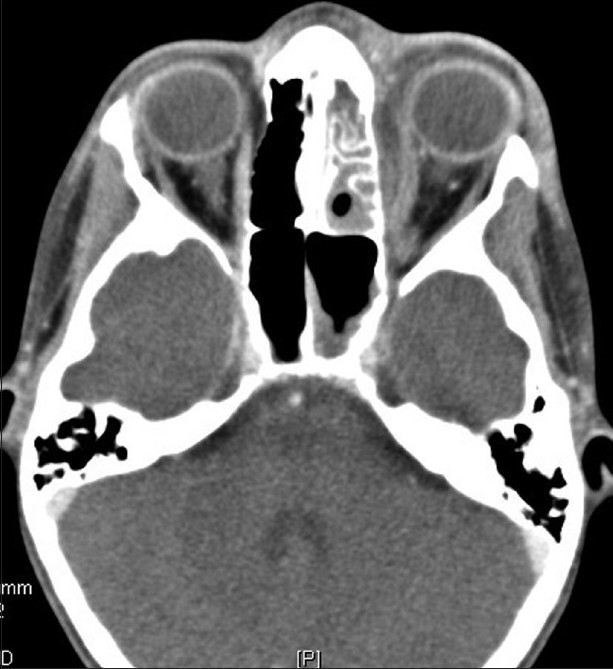
Left medial subperiosteal abscess with associated ethmoid sinusitis

Computerized tomography (CT) scan is the most commonly recommended imaging study for those suspected of having orbital cellulitis.^17^ The use of contrast media increases the sensitivity and specificity of a given study and is recommended when possible.^17^ Diffuse and localized postseptal inflammation may be observed in the setting of bacterial orbital cellulitis. Localized inflammation in the form of abscess may be intra- or extraconal. Such inflammation may also develop between the bone and periorbita, resulting in a subperiosteal abscess [Figure 2]. Radiation exposure is of concern, especially in the pediatric population and has been the subject of research.^18^ The clinical utility of a CT imaging study often out weights the risks of limited radiation exposure.^19^

## MANAGEMENT

Pediatric patients suspected of having orbital cellulitis should be admitted and appropriately studied. Broad-spectrum antibiotics should be employed to target the most common suspected pathogens. Commonly used antibiotics may include vancomycin, ampicillin-sulbactam, or piperacillin-tazobactam. Nasal decongestants are recommended given the frequency of sinusitis in these patients. Treatment should continue for at least 3 days and up to 10 days in cases of suspected or proven bacteremia.^1^

As described by Jain and Rubin, patients with orbital abscesses can be grouped into three categories of patients: (1) those requiring emergent drainage, (2) those who may need urgent drainage, and (3) those who may need expectant observation.^1^ Patients with optic nerve or retinal compromise secondary to mass-effect may require emergent drainage. Canthotomy and cantholysis may provide temporary relief, but drainage of the abscess is necessary.^1^ Those with frontal sinusitis, intracranial complications, or large subperiosteal abscesses with significant discomfort require urgent drainage. Patients younger than 9 years of age with subperiosteal abscesses may be expectantly observed. These patients may require frequent assessment of the pupillary reflex to prevent any visual loss.^1^

Research by Harris provides the basis for the last group of patients.^20^ In his study, Harris found that as humans age, their subperiosteal abscesses are more complicated in terms of organism number and type, and do not respond as well to antibiotics, and require surgical drainage more often. Patients less than 9 years of age may clear subperiosteal abscess without surgical drainage more often than their older counterparts.^20^

## COMPLICATIONS OF ORBITAL CELLULITIS

The complications of orbital cellulitis may be local or systemic. Ocular complications may include corneal disease, retinitis, uveitis, exudative retinal detachment, optic neuropathy, endophthalmitis, and globe rupture.^1^,^7^ A motility defect, intracranial disease, sepsis, and death have all been reported due to untreated orbital cellulitis.^1^,^3^,^11^ Most of the complications may resolve concordantly with the orbital cellulitis and in rare cases some may persist.

## SUMMARY

Pediatric bacterial orbital cellulitis is an infectious inflammation within the postseptal space that has been associated with significant morbidity and occasional mortality. Distinguishing preseptal from orbital cellulitis is critical to the appropriate management as is the distinction of bacterial orbital cellulitis from other postseptal processes. *Staphylococcus aureus* and *Streptococcus* species are the most common pathogens. Diagnostic tests including imaging studies should be judiciously applied that may reveal ethmoid sinusitis. Patients should be admitted and treated with broad-spectrum antibiotics and when needed surgical intervention be carried out. Monitoring for complications may prevent significant morbidity and mortality.

## References

[1] Jain A, Rubin PA (2001). Orbital cellulitis in children. Int Ophthalmol Clin.

[2] Botting AM, McIntosh D, Mahadevan M (2008). Paediatric pre- and post-septal peri-orbital infections are different diseases: A retrospective review of 262 cases. Int J Pediatr Otorhinolaryngol.

[3] Nageswaran S, Woods CR, Benjamin DK, Givner LB, Shetty AK (2006). Orbital cellulitis in children. Pediatr Infect Dis J.

[4] Cruz AA, Mussi-Pinhata MM, Akaishi PM, Cattebeke L, Torrano da Silva J, Elia J (2001). Neonatal orbital abscess. Ophthalmology.

[5] Miller A, Castanes M, Yen M, Coats D, Yen K (2008). Infantile orbital cellulitis. Ophthalmology.

[6] Wald ER (2004). Periorbital and orbital infections. Pediatr Rev.

[7] Youssef OH, Stefanyszyn MA, Bilyk JR (2008). Odontogenic orbital cellulitis. Ophthal Plast Reconstr Surg.

[8] Chandler JR, Langenbrunner DJ, Stevens ER (1970). The pathogenesis of orbital complications in acute sinusitis. Laryngoscope.

[9] Kloek CE, Rubin PA (2006). Role of inflammation in orbital cellulitis. Int Ophthalmol Clin.

[10] Rothstein J, Maisel RH, Berlinger NT, Wirtschafter JD (1984). Relationship of optic neuritis to disease of the paranasal sinuses. Laryngoscope.

[11] Ambati BK, Ambati J, Azar N, Stratton L, Schmidt EV (2000). Periorbital and orbital cellulitis before and after the advent of Haemophilus influenzae type B vaccination. Ophthalmology.

[12] Smith TF, O'Day D, Wright PF (1978). Clinical implications of preseptal (periorbital) cellulitis in childhood. Pediatrics.

[13] Schwartz GR, Wright SW (1996). Changing bacteriology of periorbital cellulitis. Ann Emerg Med.

[14] Patt BS, Manning SC (1991). Blindness resulting from orbital complications of sinusitis. Otolaryngol Head Neck Surg.

[15] Donahue SP, Schwartz G (1998). Preseptal and orbital cellulitis in childhood. A changing microbiologic spectrum. Ophthalmology.

[16] Blomquist PH (2006). Methicillin-resistant *Staphylococcus aureus* infections of the eye and orbit (an American Ophthalmological Society thesis). Trans Am Ophthalmol Soc.

[17] Eustis HS, Mafee MF, Walton C, Mondonca J (1998). MR imaging and CT of orbital infections and complications in acute rhinosinusitis. Radiol Clin North Am.

[18] Brenner D, Elliston C, Hall E, Berdon W (2001). Estimated risks of radiation-induced fatal cancer from pediatric CT. AJR Am J Roentgenol.

[19] Jaffurs D, Denny A (2009). Diagnostic pediatric computed tomographic scans of the head: Actual dosage versus estimated risk. Plast Reconstr Surg.

[20] Harris GJ (1993). Age as a factor in the bacteriology and response to treatment of subperiosteal abscess of the orbit. Trans Am Ophthalmol Soc.

